# Association between fibrinogen‐to‐albumin ratio and functional prognosis of 3 months in patients with acute ischemic stroke after intravenous thrombolysis

**DOI:** 10.1002/brb3.3364

**Published:** 2023-12-31

**Authors:** Xinxin Chen, Xiahong Xu, Ying Li, Feifeng Liu, Bei Zhang, Lian Zuo

**Affiliations:** ^1^ Department of Neurology School of Medicine Shanghai East Hospital Tongji University Shanghai China

**Keywords:** acute ischemic stroke, fibrinogen‐to‐albumin ratio, intravenous thrombolysis, prognosis

## Abstract

**Background:**

The presence of high fibrinogen and low albumin levels in serum is associated with a negative prognosis in acute ischemic stroke (AIS). Fibrinogen‐to‐albumin ratio (FAR), a new inflammatory biomarker, may provide better prognostic insights in patients with AIS than separate evaluation of fibrinogen or albumin. The objective of this investigation is to examine the correlation between FAR and 3‐month functional prognosis after intravenous thrombolysis (IVT) in AIS patients.

**Methods:**

The retrospective study recruited AIS patients who received IVT from June 2014 to December 2021. The 3‐month functional prognosis was assessed using the Modified Rankin Scale (mRS). A mRS score of ≤2 indicated a good outcome, whereas a mRS score of >2 suggested a poor outcome.

**Results:**

A total of 591 AIS patients who underwent IVT were included and 147 patients (24.9 %) had a poor outcome. Among the 102 pairs of patients after propensity score matching, there was a significant association between FAR and 3‐month prognosis (adjusted OR, 1.19; 95% CI, 1.03–1.38; *p* = .020). The optimal FAR cutoff value was found to be 7.57, and even after stratifying patients based on this value, we still observed a significant correlation between high FAR level and poor outcome (adjusted OR, 2.08; 95% CI, 1.28–3.40; *p* = .003).

**Conclusions:**

FAR may serve as a prospective biomarker of predicting 3‐month prognosis in AIS patients after IVT.

## Graphical abstract

Fibrinogen‐to‐albumin ratio may serve as a prospective biomarker of predicting 3‐month prognosis in acute ischemic stroke patients after intravenous thrombolysis. Elevated fibrinogen‐to‐albumin ratio levels enhance the probability of poor outcome in acute ischemic stroke patients after intravenous thrombolysis.

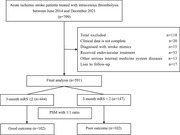

## 1 INTRODUCTION

Stroke is a primary contributor to functional impairment and imposes substantial health and economic burdens in China (Wu et al., 2019). Currently, intravenous thrombolysis (IVT) utilizing recombinant tissue plasminogen activator (rt‐PA) is the preferred modality for managing acute ischemic stroke (AIS), which significantly improving neurological prognosis (Campbell et al., 2019). However, previous research indicates that only approximately 50% of patients who receive IVT achieve favorable outcomes (Ahmed et al., 2010). The response of patients to rt‐PA treatment may vary and could be influenced by a wide range of variables, including advanced age, male gender, National Institutes of Health Stroke Scale (NIHSS) score at admission (Romano et al., 2015) and elevated blood glucose levels (Miller et al., 2011). Apart from the NIHSS score, serum biomarkers may have a greater impact on the outcomes of AIS as potential risk factors. However, previous studies have paid little attention to the role of serum biomarkers in the prognosis.

Fibrinogen (FIB) has a significant correlation with platelet activation, fibrin formation, and increased plasma viscosity (Chapin & Hajjar, 2015). Elevated levels of FIB result in increased blood flow resistance, which promotes the formation of arterial plaque and thrombus (Tousoulis et al., 2011). Additionally, FIB participates in systemic inflammatory responses, inducing cytokine and chemotactic factor secretion, and promoting the release of active oxygen species, ultimately leading to brain injury (Luyendyk et al., 2019; Petersen et al., 2018). Albumin (ALB), as a negative inflammatory biomarker, has multiple functions including antiapoptotic, antioxidant, and anti‐inflammatory activities (Oettl & Stauber, 2007). It plays a crucial role within body, including inhibiting platelet activation and aggregation, reducing neurotoxicity in patients with stroke, and scavenging free radicals to improve functional prognosis (Gabay & Kushner, 1999; Zhang et al., 2016). Besides, ALB accumulates in areas of inflammation; therefore, it can serve as an ideal drug delivery platform (Sleep, 2015). Fibrinogen‐to‐albumin ratio (FAR), a new inflammatory biomarker, may provide better prognostic insights in patients with AIS than separate evaluation of fibrinogen or albumin. In certain thrombotic diseases, such as ST‐segment elevation myocardial infarction (Karahan et al., 2016), lacunar stroke (Zheng et al., 2021), and pontine stroke (Zhai et al., 2022), elevated FAR levels have been identified as a reliable indicator of disease severity. Moreover, elevated FAR levels have been linked to complications in cerebral infarction, including hemorrhagic transformation and poststroke pneumonia (Lin et al., 2021; Yang et al., 2023).

However, it is currently unclear whether elevated FAR levels are associated with 3‐month prognosis in AIS patients after IVT. Therefore, the primary objective of the study is to elucidate the correlation between FAR and the 3‐month prognosis in patients with AIS following IVT.

## 2 MATERIALS AND METHODS

### 2.1 Study population

This study was conducted retrospectively and observed. Patients with AIS who received IVT therapy using rt‐PA within 4.5 h of symptom onset and were subsequently admitted to Shanghai East Hospital between June 2014 and December 2021 were enrolled in the study. All patients were managed in the stroke unit and received standard care. This study was approved by the Ethics Committee of Shanghai East Hospital Affiliated to Tongji University. The final analysis included participants who met the specified criteria.

Inclusion criteria:

1. Diagnosis of AIS and received IVT therapy within 4.5 h after onset;
2. Age ≥18 years old.

Exclusion criteria:

1. Clinical data is not complete;
2. Diagnosed with stroke mimics;
3. Received endovascular treatment after IVT;
4. There are other serious internal medicine system diseases that coexist before stroke.

### 2.2 Data collection

#### 2.2.1 Clinical information

The following retrospective data for each patient prior to IVT therapy was collected. All participants underwent standardized assessments of demographic characteristics (age, sex), vascular risk factors (hypertension, diabetes, dyslipidemia, atrial fibrillation, smoking status, alcohol consumption history, previous stroke occurrence), stroke severity (measured by the NIHSS score), the Trial of Org 10172 in Acute Stroke Treatment (TOAST), infarct location, door‐to‐needle time (DNT), onset‐to‐treatment time (OTT), endovascular treatment (EVT), and laboratory data. According to the NIHSS, patients were categorized into minor (0–4), moderate (5–15), moderately severe (16–20), and severe (>20) groups.

#### 2.2.2 Laboratory data

Laboratory parameters included high‐sensitivity C‐reactive protein (Hs‐CRP), fasting blood glucose (FBG), glycosylated hemoglobin (Hba1c), fibrinogen (FIB), and albumin (ALB). Measurements were conducted before IVT therapy, except for Hba1c levels, which were assessed upon admission to the inpatient department. The FIB and ALB were measured using the *Roche cobas 8000 C702* chemiluminescence immunoassay technology produced by Roche Diagnostics. The FAR can be determined using the formula: FAR = FIB/ALB. In this study, we focus on the FAR metric. However, due to its typically small values, for better understanding and interpretation purposes, we have applied a scale transformation by multiplying the values by 10^2^. This transformation does not affect the substantive content or conclusions of our model; it simply enhances its comprehensibility.

#### 2.2.3 Clinical outcomes

A trained and qualified neurologist, who was blind to the particulars of the situation, ascertained all subsequent clinical outcomes among the enrolled patients based on a 3‐month poststroke onset, via telephone consultations or outpatient follow‐up visits. Clinical outcomes were evaluated using the mRS. A mRS score of ≤2 indicated a good outcome, whereas a mRS score of >2 suggested a poor outcome (Broderick et al., 2017).

#### 2.2.4 Statistical analysis

The R software (version 4.2.3) was used for statistical analyses. The measurement data underwent statistical analysis using Student's *t*‐test and the Mann–Whitney *U* test. Results were presented as mean ± SD or median (IQR). Categorical variables were compared using either the χ^2^ test or Fisher's exact test, and reported as *n* (%). The attainment of a two‐tailed *p* value < .05 signifies statistical significance in the observed disparity. The propensity score matching (PSM) was performed exclusively for variables that exhibited a significance level of *p* < .05 in the comparative analysis using the MatchItR package. According to the PSM method, both groups’ baseline clinical data were balanced by adjusting for variables such as age, gender, stroke severity, TOAST, smoking, atrial fibrillation (AF), high‐sensitivity C‐reactive protein (Hs‐CRP), and fasting blood glucose (FBG). The propensity score was calculated by the 1:1 nearest neighbor matching method without replacement, with a caliper set at 0.05, in order to minimize any potential differences between matched pairs. The FAR between the two patient groups was conducted using binary logistic regression, while an optimal FAR cutoff for predicting clinical outcomes was determined through the utilization of a receiver operating characteristic (ROC) curve.

## 3 RESULTS

**FIGURE 1:**
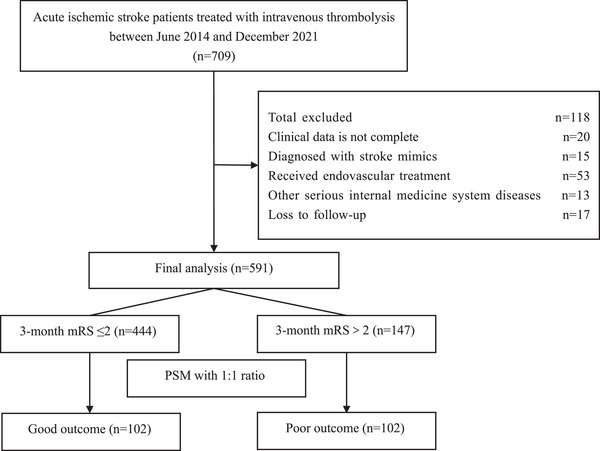
The flow chart of the selection of acute ischemic stroke patients who underwent intravenous thrombolysis.

A total of 709 AIS patients received IVT therapy from June 2014 to December 2021 were screened; 101 patients were excluded because of the exclusion criteria and 17 patients were excluded owing to an absence of follow‐up data. Finally, 591 subjects were included for analysis. Through the utilization of the PSM method, a total of 102 pairs of patients were successfully matched between the two groups. The process of selecting patients is illustrated in Figure 1.

**FIGURE 2:**
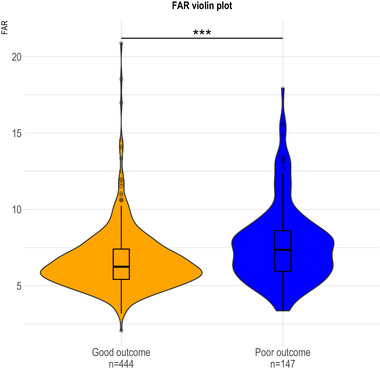
The violin plot in distribution of FAR between good outcome and poor outcome groups. ***p < .001.

Ultimately, 75.1% of patients (*n* = 444) achieved a good outcome, while 24.9% (*n* = 147) experienced a poor outcome. Based on the baseline characteristics of the participants, early baseline data categorized by good and poor outcomes were displayed in Table 1. The observed disparities between the two groups were as follows: age (*p* < .001), gender (*p* = .002), stroke severity (*p* < .001), TOAST (*p* < .001), smoking (*p* < .001), atrial fibrillation (*p* < .001), Hs‐CRP (*p* = .020), FBG (*p* = .023), FIB (*p* < .001), ALB (*p* < .001), and FAR (*p* < .001). The violin plots of FAR between the two groups were illustrated in Figure 2. The levels of FAR were discovered to be significantly elevated in patients with poor outcomes as opposed to those who experienced good outcome. In accordance with the baseline characteristics after PSM, significant differences are observed only in FIB (*p* = .009) and FAR (*p* = .016) (Table 2). The univariate logistic regression analysis unveiled a statistically significant association between FAR and clinical outcomes (OR, 1.15; 95% CI, 1.02–1.30; *p* = .024). Following adjustment for age, gender, stroke severity, TOAST, smoking, AF as well as Hs‐CRP and FBG levels in a multivariable logistic regression model, our findings indicate that FAR remains significantly linked to clinical outcomes despite accounting for these potential confounders (adjusted OR, 1.19; 95% CI, 1.03–1.38; *p* = .020) (Table 3).

**TABLE 1:** Baseline characteristics and laboratory results in patients with good and poor outcomes at 3 months.

Variable	All patients (*n* = 591)	Good outcome (*n* = 444)	Poor outcome (*n* = 147)	*p* Value
**Characteristics**				
Age (median [IQR])	68.00 [61.00, 79.00]	66.00 [59.00, 74.00]	80.00 [67.00, 86.00]	<.001*
Gender (male), *N* (%)	351 (59.4)	280 (63.1)	71 (48.3)	.002*
Stroke severity, *N* (%)				<.001*
NIHSS score (0–4)	272 (46.0)	253 (57.0)	19 (12.9)	
NIHSS score (5–15)	252 (42.6)	172 (38.7)	80 (54.4)	
NHISS score (16–20)	42 (7.1)	14 (3.2)	28 (19.0)	
NIHSS score (21–42)	25 (4.2)	5 (1.1)	20 (13.6)	
Subgroup of TOAST, *N* (%)				<.001*
Small‐artery occlusion	238 (40.3)	216 (48.6)	22 (15.0)	
Large‐artery atherosclerosis	166 (28.1)	127 (28.6)	39 (26.5)	
Cardio‐embolism	140 (23.7)	70 (15.8)	70 (47.6)	
Undetermined etiology	36 (6.1)	23 (5.2)	13 (8.8)	
Other etiology	11 (1.9)	8 (1.8)	3 (2.0)	
Infarct location, *n* (%)				.446
Anterior circulation	459 (77.7)	341 (76.8)	118 (80.3)	
Posterior circulation	132 (22.3)	103 (23.2)	29 (19.7)	
DNT, min (median [IQR])	47.0 [34.0, 70.0]	46.0 [34.0, 70.0]	49.0 [33.5, 71.0]	.568
OTT, min (median [IQR])	132.0 [96.0, 175.0]	133.0 [98.0, 180.0]	128.0 [87.5, 165.0]	.087
Hypertension, *N* (%)	415 (70.2)	303 (68.2)	112 (76.2)	.085
Diabetes, *N* (%)	153 (25.9)	107 (24.1)	46 (31.3)	.106
Smoking, *N* (%)	247 (41.8)	206 (46.4)	41 (27.9)	<.001*
Drinking, *N* (%)	105 (17.8)	84 (18.9)	21 (14.3)	.250
AF, *N* (%)	127 (21.5)	61 (13.7)	66 (44.9)	<.001*
Prior stroke or TIA, *N* (%)	90 (15.2)	60 (13.5)	30 (20.4)	.060
**Laboratory results**				
Hs‐CRP, mg/L (median [IQR])	3.00 [1.60, 5.37]	3.00 [1.60, 5.00]	3.00 [1.60, 7.90]	.020*
FBG, mmol/L (median [IQR])	6.90 [6.00, 8.80]	6.80 [5.90, 8.40]	7.30 [6.20, 10.30]	.023*
Hba1c (%) (median [IQR])	5.90 [5.60, 6.60]	5.90 [5.60, 6.60]	5.90 [5.60, 6.85]	.290
FIB, g/L (median [IQR])	2.63 [2.28, 3.12]	2.58 [2.26, 3.01]	2.81 [2.42, 3.37]	<.001*
ALB, g/L (median [IQR])	40.80 [38.00, 43.15]	41.00 [38.70, 43.50]	39.00 [36.00, 43.00]	<.001*
FAR (median [IQR])	6.46 [5.50, 7.81]	6.25 [5.42, 7.40]	7.36 [5.94, 8.61]	<.001*

*p < .05.

Abbreviations: AF, atrial fibrillation; ALB, albumin; DNT, door‐to‐needle time; FAR, fibrinogen‐to‐albumin ratio; FBG, fasting blood glucose; FIB, fibrinogen; Hba1c, glycosylated hemoglobin; Hs‐CRP, high‐sensitivity C‐reactive protein; IQR, interquartile range; NIHSS, National Institutes of Health Stroke Scale; OTT, the time from onset to treatment; TIA, transient ischemic attack; TOAST, the Trial of Org 10172 in Acute Stroke Treatment.

**TABLE 2:** Baseline characteristics and laboratory results in patients with good and poor outcomes at 3 months after PSM.

Variable	All patients (*n* = 204)	Good outcome (*n* = 102)	Poor outcome (*n* = 102)	*p* Value
Characteristics				
Age (median [IQR])	75.00 [65.00, 83.00]	74.00 [65.25, 82.00]	76.00 [63.25, 83.75]	.848
Gender (male), *N* (%)	111 (54.4)	55 (53.9)	56 (54.9)	1.000
Stroke severity, *N* (%)				.980
NIHSS score (0–4)	38 (18.6)	19 (18.6)	19 (18.6)	
NIHSS score (5–15)	132 (64.7)	67 (65.7)	65 (63.7)	
NHISS score (16–20)	26 (12.7)	12 (11.8)	14 (13.7)	
NIHSS score (21–42)	8 (3.9)	4 (3.9)	4 (3.9)	
Subgroup of TOAST, *N* (%)				1.000
Small‐artery occlusion	43 (21.1)	21 (20.6)	22 (21.6)	
Large‐artery atherosclerosis	71 (34.8)	36 (35.3)	35 (34.3)	
Cardio‐embolism	76 (37.3)	38 (37.3)	38 (37.3)	
Undetermined etiology	10 (4.9)	5 (4.9)	5 (4.9)	
Other etiology	4 (2.0)	2 (2.0)	2 (2.0)	
Infarct location, *n* (%)				.867
Anterior circulation	158 (77.5)	78 (76.5)	80 (78.4)	
Posterior circulation	46 (22.5)	24 (23.5)	22 (21.6)	
DNT, min (median [IQR])	45.00 [34.00, 65.50]	44.00 [35.00, 61.75]	45.00 [33.00, 67.00]	.834
OTT, min (median [IQR])	128.00 [89.00, 165.00]	125.50 [85.25, 166.25]	128.00 [91.25, 165.00]	.976
Hypertension, *N* (%)	149 (73.0)	72 (70.6)	77 (75.5)	.528
Diabetes, *N* (%)	62 (30.4)	26 (25.5)	36 (35.3)	.171
Smoking, *N* (%)	67 (32.8)	31 (30.4)	36 (35.3)	.551
Drinking, *N* (%)	37 (18.1)	18 (17.6)	19 (18.6)	1.000
AF, *N* (%)	73 (35.8)	36 (35.3)	37 (36.3)	1.000
Prior stroke or TIA, *N* (%)	40 (19.6)	18 (17.6)	22 (21.6)	.597
**Laboratory results**				
Hs‐CRP, mg/L (median [IQR])	3.00 [1.60, 6.00]	2.45 [1.60, 5.00]	3.00 [1.60, 7.00]	.220
FBG, mmol/L (median [IQR])	7.20 [6.10, 9.93]	7.00 [6.10, 9.57]	7.35 [6.10, 10.35]	.652
Hba1c (%) (median [IQR])	6.05 [5.60, 6.95]	6.10 [5.70, 7.02]	5.95 [5.60, 6.90]	.506
FIB, g/L (median [IQR])	2.66 [2.25, 3.18]	2.58 [2.16, 2.95]	2.78 [2.32, 3.34]	.009*
ALB, g/L (median [IQR])	40.00 [37.85, 43.00]	40.00 [38.00, 42.30]	40.00 [37.00, 43.08]	.903
FAR (median [IQR])	6.56 [5.63, 7.93]	6.27 [5.56, 7.42]	7.04 [5.76, 8.40]	.016*

*p < .05.

Abbreviations: AF, atrial fibrillation; ALB, albumin; DNT, door‐to‐needle time; FAR, fibrinogen‐to‐albumin ratio; FBG, fasting blood glucose; FIB, fibrinogen; Hba1c, glycosylated hemoglobin; Hs‐CRP, high‐sensitivity C‐reactive protein; IQR, interquartile range; NIHSS, National Institutes of Health Stroke Scale; OTT, the time from onset to treatment; PSM, propensity score matching; TIA, transient ischemic attack; TOAST, the Trial of Org 10172 in Acute Stroke Treatment.

**TABLE 3:** Binary logistic regression for the clinical outcomes at 3 months after PSM.

Variables	Univariate analysis		Multivariate analysis^a^	
	OR (95% CI)	*p* Value	OR (95% CI)	*p* Value
FAR	1.15 (1.02–1.30)	.024*	1.19 (1.03–1.38)	.020*

^a^ Age, gender, stroke severity, TOAST, smoking, AF, Hs‐CRP, FBG, and FAR were included.

*p < .05.

Abbreviations: AF, atrial fibrillation; CI, confidential interval; FAR, fibrinogen‐to‐albumin ratio; FBG, fasting blood glucose; Hs‐CRP, high‐sensitivity C‐reactive protein; OR, odds ratio; TIA, transient ischemic attack; TOAST, the Trial of Org 10172 in Acute Stroke Treatment.

**FIGURE 3:**
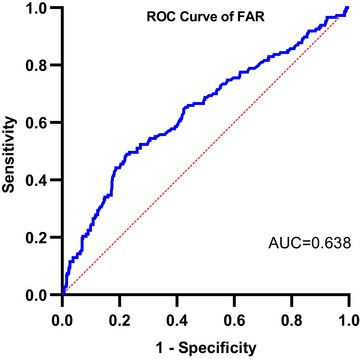
ROC analysis of FAR for predicting 3‐month functional prognosis in AIS patients after IVT. The AUC of FAR for predicting 3‐month functional prognosis is 0.638.

The analysis of the ROC curve demonstrated that the FAR exhibited an area under the curve (AUC) value of 0.638 (95 % CI, 0.58 – 0.69; *p* < .001) when predicting 3‐month prognosis in AIS patients after IVT (Figure 3). The FAR level of 7.57 was considered the optimal cutoff value for predicting clinical outcomes. The FAR demonstrated a predictive performance with a sensitivity of 48.98% and specificity of 77.48% (Table S1).

**FIGURE 4:**
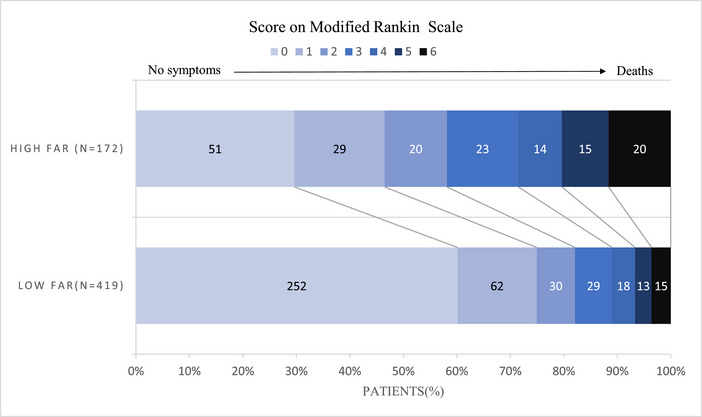
The distribution of mRS between the high FAR group and low FAR group. The mRS scores in the high FAR group were significantly higher than those in the low FAR group (*p* < .001).

To further elucidate the association between FAR levels and functional prognosis in AIS patients following IVT, all patients were stratified into two groups based on the optimal cutoff value of FAR: a low FAR group (≤7.57) and a high FAR group (>7.57) (Table 4). The distribution of mRS between these two groups is shown in Figure 4. The observed disparities between the two groups were as follows: age (*p* < .001), outcomes (*p* < .001), stroke severity (*p* < .001), TOAST (*p* < .001), AF (*p* < .001), Hs‐CRP (*p* < .001), FBG (*p* = .024), Hba1c (*p* = .034), FIB (*p* < .001), and ALB (*p* < 0.001). The relationship between FAR levels and poor outcome at 3 months was investigated through multivariate logistic regression analysis. After adjusting for age, stroke severity, TOAST, AF, Hs‐CRP and FBG, we observed an independent association between high level of FAR and poor outcome (adjusted OR, 2.08; 95% CI, 1.28–3.40; *p* = .003) (Table 5).

**TABLE 4:** Baseline characteristics and laboratory results of patients in the low (≤7.57) and high (>7.57) FAR groups.

Variable	All patients (*n* = 591)	Low FAR (*n* = 419)	High FAR (*n* = 172)	*p* Value
**Characteristic**				
Age (median [IQR])	68.00 [61.00, 79.00]	66.00 [60.00, 76.00]	73.00 [64.00, 84.00]	<.001*
Gender (male), *N* (%)	351 (59.4)	259 (61.8)	92 (53.5)	.075
Outcomes (poor outcome), *N* (%)	147 (24.9)	75 (17.9)	72 (41.9)	<.001*
Stroke severity, *N* (%)				<.001*
NIHSS score (0–4)	272 (46.0)	214 (51.1)	58 (33.7)	
NIHSS score (5–15)	252 (42.6)	170 (40.6)	82 (47.7)	
NHISS score (16–20)	42 (7.1)	24 (5.7)	18 (10.5)	
NIHSS score (21–42)	25 (4.2)	11 (2.6)	14 (8.1)	
Subgroup of TOAST, *N* (%)				<.001*
Small‐artery occlusion	238 (40.3)	187 (44.6)	51 (29.7)	
Large‐artery atherosclerosis	166 (28.1)	126 (30.1)	40 (23.3)	
Cardio‐embolism	140 (23.7)	81 (19.3)	59 (34.3)	
Undetermined etiology	36 (6.1)	20 (4.8)	16 (9.3)	
Other etiology	11 (1.9)	5 (1.2)	6 (3.5)	
Infarct location, *n* (%)				1.000
Anterior circulation	459 (77.7)	325 (77.6)	134 (77.9)	
Posterior circulation	132 (22.3)	94 (22.4)	38 (22.1)	
DNT (median [IQR])	47.0 [34.0, 70.0]	46.0 [34.0, 69.0]	49.0 [35.0, 72.3]	.369
OTT (median [IQR])	132.0 [96.0, 175.0]	132.5 [100.0, 180.5]	130.0 [89.5, 165.3]	.107
Hypertension, *N* (%)	415 (70.2)	288 (68.7)	127 (73.8)	.257
Diabetes, *N* (%)	153 (25.9)	105 (25.1)	48 (27.9)	.539
Smoking, *N* (%)	247 (41.8)	185 (44.2)	62 (36.0)	.085
Drinking, *N* (%)	105 (17.8)	80 (19.1)	25 (14.5)	.231
AF, *N* (%)	127 (21.5)	75 (17.9)	52 (30.2)	.001*
Prior stroke or TIA, *N* (%)	90 (15.2)	61 (14.6)	29 (16.9)	.561
**Laboratory results**				
Hs‐CRP, mg/L (median [IQR])	3.00 [1.60, 5.37]	2.78 [1.60, 5.00]	3.48 [1.69, 10.00]	<.001*
FBG, mmol/L (median [IQR])	6.90 [6.00, 8.80]	6.80 [5.90, 8.45]	7.35 [6.20, 9.10]	.024*
Hba1c (%) (median [IQR])	5.90 [5.60, 6.60]	5.90 [5.50, 6.60]	6.00 [5.70, 6.70]	.034*
FIB, g/L (median [IQR])	2.63 [2.28, 3.12]	2.45 [2.15, 2.68]	3.37 [3.13, 3.69]	<.001*
ALB, g/L (median [IQR])	40.80 [38.00, 43.15]	42.00 [39.00, 44.00]	38.40 [35.48, 40.70]	<.001*

*p < .05.

Abbreviations: AF, atrial fibrillation; ALB, albumin; DNT, door‐to‐needle time; FAR, fibrinogen‐to‐albumin ratio; FBG, fasting blood glucose; FIB, fibrinogen; Hba1c, glycosylated hemoglobin; Hs‐CRP, high‐sensitivity C‐reactive protein; IQR, interquartile range; NIHSS, National Institutes of Health Stroke Scale; OTT, the time from onset to treatment; TIA, transient ischemic attack; TOAST, the Trial of Org 10172 in Acute Stroke Treatment.

**TABLE 5:** Multivariate analysis of patients in the low (≤7.57) and high (>7.57) FAR groups.

	OR (95% CI)	*p* Value
Age	1.03 [1.02, 1.05]	<.001*
Stroke severity		
NIHSS score (0–4)	Reference	
NIHSS score (5–15)	1.36 [0.86, 2.15]	.192
NHISS score (16–20)	0.92 [0.39, 2.17]	.854
NIHSS score (21–42)	1.44 [0.53, 3.93]	.479
Subgroup of TOAST, *N* (%)		
Small‐artery occlusion	Reference	
Large‐artery atherosclerosis	0.82 [0.48, 1.39]	.456
Cardio‐embolism	1.56 [0.69, 3.55]	.284
Undetermined etiology	1.78 [0.77, 4.16]	.179
Other etiology	2.35 [0.61, 9.13]	.217
Poor outcome	2.08 [1.28, 3.40]	.003*
AF	0.66 [0.30, 1.47]	.308
Hs‐CRP	1.07 [1.04, 1.11]	<.001*
FBG	1.02 [0.97, 1.07]	.400

*p < .05.

Abbreviations: AF, atrial fibrillation; CI, confidential interval; FBG, fasting blood glucose; Hs‐CRP, high‐sensitivity C‐reactive protein; NIHSS, National Institutes of Health Stroke Scale; OR, odds ratio; TOAST, the Trial of Org 10172 in Acute Stroke Treatment.

## 4 DISCUSSION

Many factors are associated with the prognosis of patients who receive IVT after AIS. However, previous studies have paid little attention to the role of serum biomarkers in the prognosis. This study primarily investigates the correlation between FAR and the 3‐month prognosis.

The correlation between FAR and 3‐month prognosis of AIS patients after IVT is determined by the levels of FIB and ALB. The plasma glycoprotein FIB is synthesized by the liver and represents the most abundant coagulation factor in the human body (Levy & Goodnough, 2015). FIB is pivotal in the cascade of clot formation (Chapin & Hajjar, 2015) and is intimately linked with platelet activation, fibrin formation, and increased plasma viscosity.

Elevated levels of FIB lead to increased resistance in blood flow, reduced velocity, and the maintenance of a hypercoagulable state, ultimately promoting the formation of arterial plaques and thrombi (Tousoulis et al., 2011). In addition, FIB is involved in systemic inflammatory responses (Luyendyk et al., 2019). FIB acts as a ligand for diverse cell surface receptors, mediating cell‐to‐cell adhesion between leukocytes and endothelial cells (Yakovlev & Medved, 2018). FIB binds to receptors on astrocytes in the brain, which triggers the secretion of cytokines and chemokines, facilitating the release of reactive oxygen species, ultimately resulting in cerebral injury (Petersen et al., 2018). Elevated levels of serum matrix metalloproteinase‐9 (MMP‐9) during the acute phase of ischemic stroke are indicative of an augmented risk for mortality and significant disability (Maestrini et al., 2020; Zhong et al., 2017). FIB can activate endothelial cells, resulting in the production of MMP‐9 (Sulimai et al., 2023). MMP‐9 degrades endothelial junction proteins, compromising endothelial cell integrity, increasing cerebral vascular permeability, and ultimately causing brain tissue damage (Iadecola et al., 2020). Furthermore, FIB can directly increase the permeability of the blood‐brain barrier (BBB) by acting on cerebral endothelial cells, disrupting the integrity of the BBB, and subsequently causing neuroinflammation and neuronal damage (Petersen et al., 2018). The utilization of FIB depletion agents in patients with AIS can help mitigate neurological impairment and enhance their overall quality of life (Chen et al., 2018).

ALB, as a negative inflammatory biomarker, exhibits various functions such as antiapoptotic, antioxidant, and anti‐inflammatory effects (Oettl & Stauber, 2007). It plays a vital physiological function within the body, specifically by inhibiting platelet activation and aggregation (Gabay & Kushner, 1999). High levels of albumin can decrease neurotoxicity and scavenge free radicals, thereby improving functional outcomes in patients with AIS (Zhang et al., 2016). Conversely, hypoalbuminemia is related to adverse functional outcomes in ischemic stroke (Dziedzic et al., 2004). ALB can effectively reduce infarct volume and cerebral edema while decreasing BBB permeability in ischemic stroke (Park et al., 2017). Studies have been conducted on the use of intravenous ALB infusion to improve outcomes in patients with AIS (Huang & Xiao, 2021). However, the specific clinical results remain unclear. Moreover, ALB accumulates at the site of inflammation; therefore, it can serve as an ideal platform for drug delivery (Sleep, 2015).

The FAR is an emerging systemic inflammatory biomarker derived from the levels of fibrinogen and albumin. It has the potential to be more effective than measuring FIB or ALB individually in predicting prognosis following IVT. ALB interacts with FIB, leading to impaired activity of FIB (Galanakis, 1992). Elevated levels of FAR indicate higher levels of FIB or lower levels of ALB. Elevated levels of FAR have been identified as markers of disease severity in certain thrombotic conditions, such as ST‐segment elevation myocardial infarction, lacunar stroke, and pontine stroke (Karahan et al., 2016; Zhai et al., 2022; Zheng et al., 2021). Besides, current research has established a correlation between elevated levels of FAR and cerebral infarction complications, such as hemorrhagic transformation following thrombolysis and poststroke pneumonia (Lin et al., 2021; Yang et al., 2023), which can impact patient outcomes.

This study investigates the association between FAR and functional prognosis of 3 months after IVT in patients with AIS. First, our study findings indicate that patients with poor outcome had significantly higher baseline levels of FAR compared to those with good outcome. After adjusting for potential confounding variables, we observed an independent correlation between FAR and 3‐month prognosis. Second, the analysis of the ROC curve indicated that a FAR level of 7.57 was identified as the optimal cutoff value for predicting poor outcome occurrence. In addition, when stratifying AIS patients based on FAR's optimal cutoff value, we found that the high FAR group had a larger portion of poor outcome than the low FAR group. After adjusting for potential confounding factors, we observed an independent connection between higher levels of FAR and the occurrence of poor outcome. Consequently, our study has revealed an independent correlation between FAR and 3‐month prognosis in AIS patients after IVT. Patients with elevated FAR levels exhibit more severe neurologic deficits and have poorer outcome.

Our research may be subject to certain limitations. First, our study was based on a retrospective database from a single center, which had limited sample size and regional restrictions, potentially introducing unavoidable subjective selection bias. Further validation through multicenter and large‐sample studies is warranted. Second, our FAR data were obtained from pre‐IVT measurements, without further follow‐up to observe the changes in the ratio after IVT. Third, no nutritional assessment was conducted upon admission, and the nutritional status could potentially influence the changes in the FAR, which may be associated with the prognosis of cerebral infarction.

## 5 CONCLUSION

In summary, FAR may serve as a prospective biomarker of predicting 3‐month prognosis in AIS patients after IVT. Elevated FAR levels enhance the probability of poor outcome in AIS patients after IVT. The confirmation of these findings regarding the prognosis of patients with AIS after IVT necessitates further multicenter longitudinal studies.

## AUTHOR CONTRIBUTIONS

**Xinxin Chen**: Data curation; formal analysis; resources; software; writing—original draft. **Xiahong Xu**: Conceptualization; data curation; writing—original draft. **Ying Li**: Data curation. **Feifeng Liu**: Conceptualization; writing—original draft. **Bei Zhang**: Conceptualization; funding acquisition; software; writing—review and editing. **Lian Zuo**: Conceptualization; data curation; formal analysis; methodology; supervision; writing—review and editing

## CONFLICT OF INTEREST STATEMENT

No financial or other conflicts of interest have been disclosed by the authors.

### PATIENT CONSENT STATEMENT

Informed consent was obtained from the participants or their legal representatives.

### PEER REVIEW

The peer review history for this article is available at https://publons.com/publon/10.1002/brb3.3364.

## Supporting information

Supporting Information

## Data Availability

The datasets utilized and examined in this investigation can be acquired upon a reasonable request directed to the corresponding author.
