# Supplementary material for: Association between fibrinogen‐to‐albumin ratio and functional prognosis of 3 months in patients with acute ischemic stroke after intravenous thrombolysis
Source: Brain Behav. 2023 Dec 31;14(1):e3364. doi: 10.1002/brb3.3364 (PMC10757894; doi:10.1002/brb3.3364)
Supplement: Supplementary file 1 — Supporting Information [file BRB3-14-e3364-s001.docx]

Table s1 The receiver operating characteristic (ROC) curve of FAR.

|  | AUC | 95% CI | optimal cutoff value | specificity | sensitivity | Youden index |
| --- | --- | --- | --- | --- | --- | --- |
| FAR | 0.638 | 0.5842 - 0.6922 | 7.57 | 77.48 | 48.98 | 0.265 |
